# Epigenetic Mechanisms Induced by *Mycobacterium tuberculosis* to Promote Its Survival in the Host

**DOI:** 10.3390/ijms252111801

**Published:** 2024-11-02

**Authors:** Shwetha Susan Thomas, Kuniyil Abhinand, Arjun M. Menon, Bipin G. Nair, Geetha B. Kumar, K. B. Arun, Lekshmi K. Edison, Aravind Madhavan

**Affiliations:** 1 School of Biotechnology, Amrita Vishwa Vidyapeetham, Amritapuri, Kollam 690525, Kerala, India; 2 Department of Life Sciences, CHRIST (Deemed to be University), Bangalore 560029, Karnataka, India; 3 Department of Comparative, Diagnostic, and Population Medicine, College of Veterinary Medicine, University of Florida, Gainesville, FL 32610, USA

**Keywords:** epigenetics, tuberculosis, miRNA modification, macrophage polarization, autophagy, apoptosis

## Abstract

Tuberculosis caused by the obligate intracellular pathogen, *Mycobacterium tuberculosis*, is one among the prime causes of death worldwide. An urgent remedy against tuberculosis is of paramount importance in the current scenario. However, the complex nature of this appalling disease contributes to the limitations of existing medications. The quest for better treatment approaches is driving the research in the field of host epigenomics forward in context with tuberculosis. The interplay between various host epigenetic factors and the pathogen is under investigation. A comprehensive understanding of how *Mycobacterium tuberculosis* orchestrates such epigenetic factors and favors its survival within the host is in increasing demand. The modifications beneficial to the pathogen are reversible and possess the potential to be better targets for various therapeutic approaches. The mechanisms, including histone modifications, DNA methylation, and miRNA modification, are being explored for their impact on pathogenesis. In this article, we are deciphering the role of mycobacterial epigenetic regulators on various strategies like cytokine expression, macrophage polarization, autophagy, and apoptosis, along with a glimpse of the potential of host-directed therapies.

## 1. Introduction

All chromosomal alterations that change gene expression without changing the nucleotide sequencing of the coding DNA are considered epigenetic regulations [1]. The transcriptional profile of genes linked to the immune system is regulated via epigenetic mechanisms which facilitate the interaction between the pathogen and the host [2]. Epigenetic reprogramming is the process by which infectious agents frequently alter epigenetic processes to impact human responses related to immunity and inflammation. Promising targets for this epigenetic regulation are the host’s genes implicated in immunity, inflammation, senescence, survival, etc. [3]. Recent research has documented epigenetic modifications brought about by the interaction of the *Mycobacterium tuberculosis* (*Mtb*) pathogen with the host’s cells, which includes modification of histone, miRNA-assisted regulation, and DNA methylation [4]. *Mtb* is considered a significant immunomodulator influencing the host responses and results in epigenome alterations, affecting the transcriptional machinery [5]. Throughout human immune system evolution, *Mtb* has acted as a host as well as a reservoir. Understanding the genes necessary for its development and survival can therefore provide related information about its virulence and host defense [6]. Current research indicates that *Mtb* can modify the host epigenome to control transcriptional machinery via the activation or inhibition of significant immune genes linked to pathogen survival or immune response [7]. Current research suggests that *Mtb* can modify the host epigenome to control transcriptional machinery via activating or inhibiting significant immune genes linked to pathogen survival or immune response [7]. The traits associated with *Mtb* to negatively affect the host includes various strategies like the alterations in phagocytosis, apoptosis, autophagy, presentation of antigens, and polarization of macrophages [8]. With the ability to connect the disparities between *Mtb*, the host, and the niche, epigenetics hold considerable promise for anticipating the onset of tuberculosis outbreaks [9]. Since the existing antibiotics fail to challenge this globally threatening organism, novel approaches like host-directed therapies are a crucial need [10]. According to the WHO Global Tuberculosis Report 2023, in order to meet the targets for tuberculosis prevalence reductions, its incidence rate had to decrease at a rate of 10% annually by 2025, with an average annual rate of 17% from 2025 until 2035 [11]. Here, in this review, we have attempted to provide a summary of the recent research insights into the role of various epigenetic alterations induced by *Mycobacterium tuberculosis* to accomplish the onset of tuberculosis by combating the host immune response.

## 2. Unraveling the Mycobacterial Epigenetic Tools

**Figure 1:**
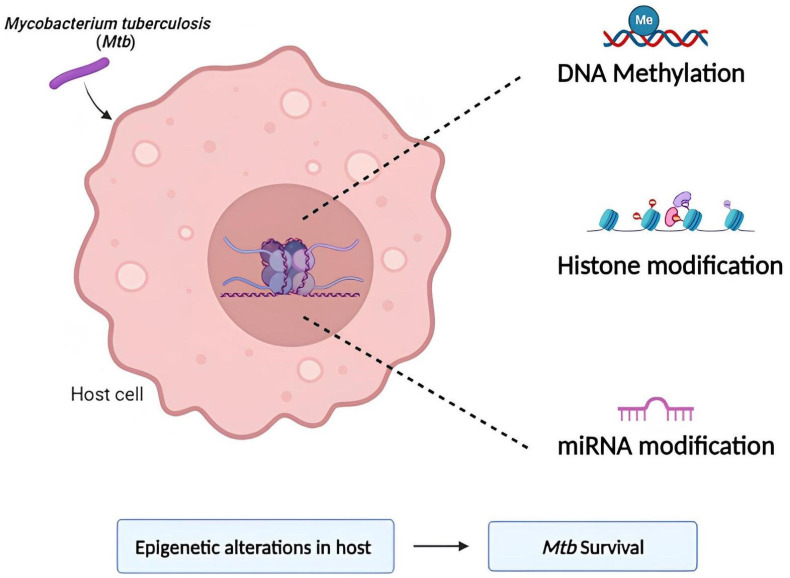
The various epigenetic alterations induced by *Mycobacterium tuberculosis*.

Among the crucial mycobacterial traits that allow for efficient host conquest, some are epifactors [12]. *Mycobacterium tuberculosis* (*Mtb*) aids to ensure self-survival within the host by functioning as an epigenetic modulator. The bacteria use several techniques, such as non-coding RNA-mediated silencing, histone and chromatin remodeling, and DNA methylation, in this context [3], and are indicated in Figure 1.

### 2.1. Histone Modification

Histone alterations and chromatin dynamics are important modulators of eukaryotic transcription; thus, they are becoming obvious targets for infections by pathological agents [13]. Chromatin formation begins with the octamer of four basic histones, H2A, H2B, H3, and H4, being wrapped around 147 nucleotide combinations of DNA. The dynamic characteristic of chromatin is also conversed by histone-modifying enzymes. In fact, phosphorylation, acetylation, methylation, and ubiquitylation are some of the covalent changes that can occur, and these modifications primarily affect the N-terminal tails of the histones but are also possible within the core [13]. The removal of these dynamic modifications is catalyzed by factors known as epigenetic erasers. To assess the effective modifications existing at an exact location on a specific histone, a remarkable balance between these enzymes is crucial [14]. ESAT-6 (Early Secreted Antigen-6), the secretory protein of *Mtb*, has been shown to cause acetylation at the promoter of CIITA (class II trans activator gene) and histone methylation at the H3K4 location, which controls the release of IFN-γ [15]. The function of several *Mtb*-specific proteins in histone methylation has been investigated. A few of these proteins with well-defined roles include Rv1198, Rv1988, SET8, and RV2966c [2,16]. The modification of host histone proteins by Rv2966c and Rv1988 is a crucial factor in the abduction of the host immune system and its subsequent modifications. Rv1988 works via changing the expression of genes that are crucial for the formation of ROS, such as *NOX1*, *NOX4*, *NOS2*, and *TRAF3*, which produce type I IFN [2]. Sharma et al. determined that the host cell’s cytoplasm as well as its nucleus are niche to the *Mtb* protein Rv2966c, a DNA methyltransferase specific to 5-methylcytosine. This statement is supported by the inhibition of host genes after *Mtb* infection, which is linked to the binding of Rv2966c and non-CpG methylation [16]. H3K27, the host histone protein, acquires hypermethylation because of the suppression of the *KDM6B* gene by *Mtb* [4]. Enzymes called histone acetyltransferases (HATs) attach acetyl groups to the lysine residues of host proteins, including histones, as well as transcription factors [2]. Several host enzymes, including matrix metalloproteinase (MMP), orchestrate the inflammatory reaction against the *Mtb* [4]. Bacterial intracellular survivability in the host is influenced by the acetylation of MMP-1 and MMP-3, which breaks down the lung’s extracellular matrix. While inside the nucleus of infected macrophages, Rv3423.1 acetylates histone H3 at the K9/K14 sites. This protein may play a critical role in both the pathogenicity and survival of *M. tuberculosis*, since the researchers observed that it was only found in the suspension filtrate of virulent strains of the bacteria, not in avirulent ones [2]. It is well known that HDACs are negative modulators of gene expression; however, HAT-mediated histone tail acetylation increases the gaps between nucleosomes, thereby activating chromatin [17]. Histone deacetylases such as HDAC1 and HDAC3 along with Sirtuin (SIRT1 and SIRT2) are positively and negatively modulated, respectively, to influence various pathways to encourage bacterial survival [18]. Substantial studies are presently being executed to assess the therapeutic efficacy of various HDAC inhibitors in bacterial infections [19]. To ensure proper control over gene expression, maintaining balance in histone modification is vital.

### 2.2. DNA Methylation

The most extensively researched epigenetic marker is DNA methylation. According to Chen et al., diverse levels of DNA methylation across the *PARP9*/*miR505*/*RASGRP4*/*GNG12* genes could influence the start of active tuberculosis [9,20]. A CpG site, often known as an island, is a phosphate linkage formed by a higher proportion of CG dinucleotide sequence and is considered the site of DNA methylation [3]. This mechanism is a persistent epigenetic modification that prevents the attachment of transcription factors, thereby leading to suppressed gene expression and a lack of adequate immune responses [21]. By increasing the methylation of specific genes involved in immune activation, the bacteria can suppress the immune response. On the other hand, hypomethylated genes also benefit *Mtb*, which shows the multiple strategies that can be taken [22]. To suppress transcription, DNA methylation enrolls methyl-CpG binding domain proteins, namely, MeCP2, MBD1, MBD2, MBD3, and MBD4, thereby activating histone deacetylases to suppress transcription [23]. The methylation patterns of THP-1 monocytes and monocyte-derived macrophages throughout their entire genomes have revealed that *Mtb* infection disrupts host gene expression through mechanisms reliant on changes in DNA methylation [18]. In an investigation, BCG responders’ PBMC showed increased antimycobacterial activity of MDM and variable methylation of many gene promoters, which includes *IFN-γ*, *RASAL1*, *TLR6*, and *NFKBIE*, for a period of 4 months [24]. Alveolar macrophages of some people have either an epigenetically predisposed immunological response to *Mtb* or their DNA methylation is aimed at before the adaptive immune response is triggered. Exposure to *M. tuberculosis* elicits more significant epigenetic modulations in pulmonary immune cells when weighed against peripheral blood monocytes [25].

As DNA methylation is a mechanism exhibited by various chronic infectious agents to escape from host’s immune responses, extensive studies have been executed in this context in tuberculosis. The DNA methylation profile of *Mtb*-infected patients has displayed excessive methylation in TNF/NF-κB, IFN-γ, and IL-2/STAT5 signaling pathways [26]. The attachment of a methyl group from S-adenosyl methionine (SAM) onto the cytosine (C) residue of the CpG sequence found in the genome is a tightly controlled process [2]. Mam A is a mycobacterial DNA methyl transferase that is required for *Mtb* replication. It may incorporate N6-methyladenine into a recognition sequence present in the *Mtb* genome, conferring survival advantage to the pathogen [27]. The tumor suppressor protein CD82 is an additional instance of DNA methylation having a significant impact on tuberculosis development. A study claims that the RUNX1-Rab5/22 transcription factor causes CD82 to undergo epigenetic reprogramming after infection with *Mtb*. CD82′s interaction with this transcription factor requires hypomethylation. As a result, an increased level of both CD82 and transcription factor is preferable and has been examined in the development of granulomas [28]. Major shifts in the methylation levels of genes associated with inflammation are caused by *Mtb*-infected macrophages, with the promoter region of IL-17 exhibiting a greater rise than other receptors in infected macrophages. Furthermore, the host genotype and nature of the *Mtb* strain influence these methylation pattern alterations [29].

### 2.3. miRNA Expression

MicroRNAs, the commonly observed non-coding RNAs, have been identified as post-transcriptional modulators. Many of these impact the microbe’s abilities to multiply, undergo apoptosis, respond to both pro- and anti-inflammatory stimuli, and to reside in the host [3]. Their usual length is between 20 and 22 nucleotides [30]. Post-translational changes like phosphorylation and sumoylation control miRNA synthesis [31]. The miRNAs bind to specific sequences in the 3′ untranslated region of their target mRNA, which can either break down the mRNA or prevent its translation [32]. Proper miRNA expression is essential for preserving homeostasis. Moreover, changes in cellular miRNA levels driven by natural or artificial means could have profound effects on life. Epigenetic changes in the human genome are an extensive cause in this context. These alterations not only impact cellular functions but also lead to potentially fatal disease conditions. Additionally, mycobacteria alter miRNAs linked to signaling pathways to improve their survival within hosts [33]. Together with other intricate epigenetic processes, including chromatin remodeling and genome organization within the nucleus, these miRNAs control key cellular pathways like cell division, angiogenesis, and invasion [34]. The miRNA mediates several signaling pathways and autophagy during *Mtb* infection. In this regard, overexpression of miR-1178 as well as miR-708-5p adversely affects TLR-4, which in turn inhibits the expression of inflammatory cytokines including IFN-γ, IL-6, IL-1, and TNF-α [4]. A single mRNA can be targeted by many microRNAs, and each miRNA has the potential to silence multiple genes. Consequently, disease-specific miRNAs constitute a novel class of therapeutic targets or diagnostic indicators [35]. The escalation of drug efflux, transition in targets, suppression of apoptosis, and accelerated DNA damage repair are all factors influencing drug resistance [36]. The miRNAs have an impact over these processes, including cell cycle, proliferation, apoptosis, and immunological response [37,38], and any significant alteration in miRNAs can thereby effect drug resistance [39]. *Mtb* modifies miRNAs at the molecular level, which may hijack cell differentiation and tailor the macrophage responses to ensure their survival [40]. Overexpression of miR-21-5p has been observed to suppress the TLR2/TLR1-linked antimicrobial activity against *Mtb*. However, the activity can be restored by downregulating the same [41]. Similarly, mycobacterial survivability has been enhanced by the elevation of miR-26a and miR-132, triggered by live and attenuated *Mtb*, which adversely influences the p300 mRNA within human monocyte-derived macrophages [42].

A study revealing an elevated level of over 59 miRNAs in the serum of tuberculosis patients in contrast to healthy controls demonstrated the significance of miRNAs in patients with *Mtb* infections [43]. One of the extensively studied miRNAs in tuberculosis is miR-155-5p, which has been shown to be altered by infection [35]. One literature survey indicates that miR-155-5p can be host-beneficial, enhancing the survival of *Mtb*-specific T cells as well as harmful for the host via autophagy suppression [22]. MiR-125b inhibits TNF production, while miR-29 targets INF-γ to regulate immune responses against *Mtb*. Because of its association with the clinical manifestation of the disease, miR-29 has been proposed as a diagnostic for pulmonary tuberculosis [35,44]. *Mtb* was discovered to downregulate the levels of miRNA *let-7f*, a gene that targets the mRNA of A20, an NF-κB inhibitor. Remarkably, in mice infected with Mtb, there is a simultaneous overexpression of A20 and a downregulation of *let-7f* [45]. Numerous studies have documented the distinct expression patterns of miRNAs in various cell types upon mycobacterial infection. Understanding the specific roles of these miRNAs is of great interest, as it not only enhances our knowledge but also holds promise for the generation of authentic biomarkers for the diagnosis of tuberculosis [35].

## 3. Mycobacterial Epigenetic Regulation of Innate Effectors

### 3.1. Cytokine Expression

By resisting the host’s immune system, *Mycobacterium tuberculosis* (*Mtb*) has the potential to become a chronic infection. Because of its mutual evolution with humans, *Mtb* has developed several strategies for subverting the host’s immune systems [46]. Cytokines are always released when bacteria and the host cells interact; the specific cytokines released depend primarily on the type of bacteria as well as the host cells affected [47]. Macrophage phenotype in response to infectious conditions is determined by epigenetic change in macrophage genes, which influences cytokine secretion [48]. It is well recognized that immunosuppressive cytokines, such as IL-10, influence immune cells and encourage *Mtb* infection [49]. Experiments in macrophages showed that when HDAC6 expression declined and HDAC11 expression increased, the level of IL-10 was reduced, suggesting the former is a transcription activator and the latter a suppressor of IL-10 [50]. During *Mtb* H37Rv infection, IL-10 production due to acetylation and CCR5/ERK-regulated histone phosphorylation inhibited MHC-II expression within macrophages [49]. TNF-α is undoubtedly the most well-known human immune factor that combats mycobacteria. Thus, *Mtb* could overcome the host’s battle against tuberculosis by expressing specific mycobacterial proteins and reducing the synthesis of TNF-α [51]. Rajaram et al. demonstrated how *Mtb* altered cytokine production by epigenetically changing the gene expression of the cytokine, consequently aiding in the pathogenesis [52]. On infecting macrophages with TB-LM (Lipomannan) and live *Mtb*, increased expression of *miR-125b* and decreased expression of *miR-155* and *TNF-α* release were observed [52]. Human monocytes incubated with *Mtb* produced less IL-1β as well as IL-8 than those treated with an equivalent quantity of BCG vaccine. This shows that *Mtb* can cause depleted synthesis of IL-1β and thus conveys that it can inhibit the generation of inflammasomes [53]. Suberoylanilide hydroxamic acid (SAHA) is an HDAC inhibitor that promoted the first transition to glycolysis, boosted the levels of IL-1β, and reduced IL-10 release in *Mtb*-infected macrophages. SAHA treatment exhibited increased pro-inflammatory activity. Immune-metabolic pathways in human macrophages can be altered by this inhibitor, leading to an increase in pro-inflammatory reactions [54].

The miR-146a targets TNF receptor-associated factor-6 (TRAF-6) and interleukin-1 receptor-associated kinase-1 (IRAK-1) as two important factors associated in the TLR/NF-κB signaling pathway cascades. It is probable that the elevated expression of miR-146a during *Mtb* infection may impact these pathways, followed by a decline in the synthesis of pro-inflammatory cytokines, which includes TNF-α, IL-1β, IL-6, and also the chemokine MCP-1 [33]. Through the elevation of the levels of miR-223, Mtb can downregulate the levels of CXCL2, CCL3, and IL-6 [55]. According to a recent investigation, miR-27a inhibits the immune response in *Mtb* infection by focusing on IRAK4. After miR-27a mimics were transfected, there was a substantial drop in the levels of TNF-α, IL-6, IFN-γ, and IL-β [56]. Similarly, in the case of the EIS protein (Enhanced Intracellular Survival), its N-acetyltransferase domain is responsible for the alterations in ROS production along with the synthesis of pro-inflammatory cytokines via the JNK pathway [57]. Studying cytokine expression and the impact of epigenetics over it can provide relevant insights into the regulation of gene expression and the host’s immune responses (Table 1). This can be a promising approach for understanding and implementing specific treatment regimes.

### 3.2. Macrophage Polarization

**Figure 2:**
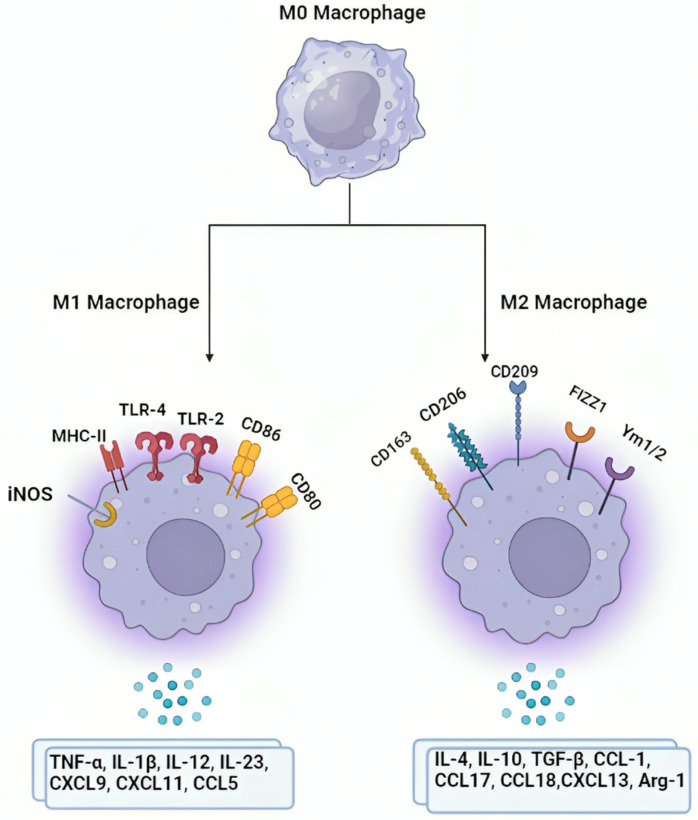
An overview of macrophage polarization.

Another vital host immune evasion strategy exhibited by *Mtb* is macrophage polarization (Figure 2). Macrophage polarization is a fascinating concept in which macrophages display a specific functional reaction to the surrounding milieu [58]. In order to promote the spread of infection, mycobacteria associate with macrophages and alter their polarization status. Therefore, knowledge of the pathophysiology of mycobacterial infections and the identification of treatment targets depends primarily on the features of macrophages in these conditions [48]. After being subjected to cytokines or microbial stimuli, circulating monocytes from the bone marrow develop into macrophages (M0s) at infection sites. *Mycobacterium tuberculosis* (*Mtb*) infects and develops in naive M0s during the course of the disease [59]. Upon mycobacterial infection, macrophages become activated due to changes in bioenergetic pathways, variable cytokine production, and epigenetic alterations of genes. These approaches promote macrophages into M1 or M2 [60]. Present theories about macrophage plasticity suggest that pro-inflammatory stimuli like LPS and IFN-γ direct the M1 phenotype to increase the production of cytokines that induce inflammation. Consequently, a pro-inflammatory phenotype is established that facilitates the antimicrobial M1 profile to be programmed quickly. The M2 phenotype is programmed by anti-inflammatory cytokines like TGF-*β*, IL-10, and IL-13, which induce the cell to release an increased number of anti-inflammatory cytokines [61]. Figure 2 represents an outline of the macrophage polarization. An overlap of genes regulated by Mycobacterium tuberculosis and IFN-γ, equivalent to an M1 phenotype, is revealed via the preliminary transcriptome investigation of mouse macrophage feedback [62].

In the case of tuberculosis patients, the M1 phenotype was more observed in non-granulomatous conditions, whereas the M2 phenotype was observed in others [63]. HMGN2 (high-mobility group N2), a non-histone nuclear protein, was discovered to be produced during the polarization of M1 macrophages stimulated by non-tuberculous mycobacteria. Research findings indicate that HMGN2 deficiency in such infected macrophages stimulates M1 markers and nitric oxide generation through a greater stimulation of MAPK and NF-κB signaling [64]. Existing studies prove that ESAT-6, a secretory protein of *Mtb*, promotes differentiation of M0 macrophages and switches the M1 phenotype to the anti-inflammatory M2 phenotype [65]. Gaining insights into the process of the paradoxical concept of macrophage polarization will broaden our comprehension of its underlying mechanisms [61].

### 3.3. Autophagy

Autophagy is a critical homeostatic mechanism that can be epigenetically altered by a variety of intracellular microbes, including *Mtb* [49]. Aravind et al. investigated a complex where HDAC1 (histone deacetylase 1) gets associated with ZBTB25 (Zinc finger and BTB domain 25), a transcription repressor, along with Sin3a, a corepressor. Upon *Mtb* infection, it has been observed that this complex binds to the promoter of *IL-12b*, thereby suppressing the transcription in macrophages [66]. Moreover, IL-12 also stimulates the JAK/STAT pathway [67]. Therefore, the aforesaid suppression negatively affects the activation of this pathway, preventing the elimination of *Mtb*. Administering HDAC1 and ZBTB inhibitors to macrophages has enhanced the JAK2/STAT4 phosphorylation, conferring its role in autophagy [66].

According to several researchers, *Mtb* exploits the control of miRNAs or lncRNAs as a key survival approach within host cells [68]. Upregulation of miR-30A inhibits autophagy, which in turn results in a lack of clearance of intracellular *Mtb*. Therefore, miR-30A is a promising therapeutic target for tuberculosis treatments [69]. Similarly, in macrophages infected with Mtb, the autophagy was enhanced by the suppression of miR-23a-5p, whereas the process was inhibited by its upregulation. Direct miRNA association to TLR2 is the mechanism via which miR-23a-5p influences TLR2 expression. Reduced TLR2 expression and corresponding TLR2/MyD88/NF-κB activity are brought about via the upregulation of miR-23a-5p [70]. By specifically targeting DRAM2, it has been discovered that miR144* serves a significant role in inhibiting the maturation of phagosomes as well as antimicrobial activities in host macrophages challenged with *Mtb* [71]. The miR-155 has also been studied widely in this aspect. Through the activation of miR-155, *Mtb* decreases the amount of the ATG3, which is essential during the commencing stages of autophagy [72]. In contrast to patients without infection, those with latent tuberculosis infection showed higher expression levels of miR889. TWEAK, or tumor necrosis factor-like weak inducer of apoptosis, was determined to be the target of miR889. To sustain mycobacterial existence within granulomas, miR-889 suppressed TWEAK expression post-transcriptionally, inhibiting autophagy [73]. In the presence of *Mtb*, miR-17 is downregulated, whereas its targets, Mcl-1 and STAT-3, are elevated concurrently. Contrary to this, if miR-17 is compelled to express, it can also impact the interplay between Mcl-1 and Beclin-1. The miR-17 has been observed to target the negative modulators of autophagy. Upon infected macrophages, overexpression of miR-17 inhibits the PKCδ phosphorylation. While the exact pathway remains elusive, the involvement of miR-17 in autophagy also exhibits the significance of epigenetics [74]. The miR26A has also been reported to have influence on autophagy modulation by aiming at Mcl-1. Additionally, enhanced levels of miR-426-5p inhibit the fusion of autophagosome and lysosome in tuberculosis conditions [75].

Another study was conducted on the *Mtb* protein, EIS (Enhanced Intracellular Survival), and its role over autophagy. Studies revealed that this protein has a crucial role in modulating host innate reactions in a ROS-dependent pathway. EIS is also critical for controlling inflammatory reactions in macrophages as well as the early production of reactive oxygen species. The acetyltransferase moiety of the protein is what is responsible for these mechanisms [76]. The degree of histone H3 acetylation was markedly increased by this protein, EIS. The interaction was observed in the SP1 and STAT3 areas of the *IL-10* promoter region. Consequently, the protein might have regulated the promoter of histone H3 acetylation in order to increase the transcription of the *IL-10* gene. They found that the autophagy initiated by rapamycin was suppressed by EIS, whereas the *IL-10* expression stimulated the Akt/mTOR/p70S6K pathway [57]. BRD4 (Bromodomain containing 4), a histone acetylation reader, has been induced to be expressed by *Mtb* via the aid of Epidermal Growth Factor Receptor (EGFR) signaling. Lipid-specific autophagy has been observed to be suppressed under this mechanism. Increased autophagic flux was resulted when Egfr or Brd4 was knocked down [77]. Analyzing the impact of epigenetics on autophagy is an intriguing research area (Table 2). By delving into specific mechanisms, better understanding can be gained over the interplay between mycobacteria and the host’s autophagic machinery.

### 3.4. Apoptosis

As an intrinsic defense mechanism, controlled apoptosis of cells is crucial for maintaining organismal homeostasis [78]. In this regard, potent anti-apoptotic systems are essential for intracellular infections to persist [79]. After the *Mtb* endures and multiplies within the host cell, the infected macrophages ultimately undergo two prevalent cell death mechanisms which include apoptosis as well as necrosis [80]. There exists a paradigm suggesting that the apoptotic death favors the host, whereas the necrotic death is advantageous to the bacteria. However, this paradigm lacks conclusive evidence, and more scientific outcomes must be generated to support this notion [81]. Macrophage apoptotic inhibition is considered a major survival mechanism exhibited by pathogenic *Mtb* to maintain a niche for replication, providing deteriorating impacts over the host cells [80]. Many intracellular pathogens are often destroyed via a process known as efferocytosis, the engulfment of apoptotic cells. The underlying mechanism of bacterial effectors comprises the attachment of secreted proteins and their inhibitory activities against the signaling pathways associated with apoptosis [78]. Macrophage apoptosis can suppress the infection induced by mycobacteria via triggering innate as well as adaptive immune responses. When compared with highly virulent mycobacteria like *Mtb* H37Rv, moderately virulent strains like BCG and the innocuous strain *Mtb* H37Ra are more effective at inducing macrophage apoptosis [82].

A histone methyl transferase called SET 8 or particularly histone H4 lysine 20 monomethylase (H4K20me1) functions as an epigenetic modulator of NQO1 (NADPH dehydrogenase quinone 1) and TRXR1 (thioredoxin reductase). This protein, in association with FoxO3a, leads to an interplay among NQO1 and PGC1-α, which not only assists in macrophage polarization to the M2 state but also promotes TRXR1-mediated apoptotic halt [49,83]. *Mtb* PE17 or Rv1646 functions through chromatin remodeling via H3K9me3. Through this, it can modulate macrophage apoptosis [84]. *Mtb* employs an enzyme complex, NADH dehydrogenase 1 (NDH-1), generally required for energy synthesis to combat the release of ROS by the host cell via the control of the NADPH oxidase-2 (NOX-2) enzyme. *Mtb* mutant strains lacking this complex were observed to show increased abundance of ROS within the macrophage, thereby secreting TNF-α, leading to enhanced inflammation and apoptosis [85]. PPARγ, or peroxisome proliferator-activated receptor γ, is a global transcriptional modulator known with anti-inflammatory properties. Using PPARγ, *Mtb* suppresses apoptosis through the modulation of Mcl-1 and Bax, where the former is considered a pro-survival factor and the latter is pro-apoptotic. 15-lipoxygenase (15-LOX) is essential for the aforesaid activity [86].

The miRNAs are considered critical on this aspect as well. A negative modulator of apoptosis in tuberculosis is miR-20a-5p. Reduced levels of this can lead to apoptotic conditions, thereby initiating the mycobacterial clearance from the host, where JNK2 is the target [87]. For apoptosis initiation, miR-20b-5p considers Mcl-2 as the target and Bcl2 for miR-21-5p. The upregulation of miRNA-1281 also protects hosts’ macrophages, where it targets Cyclophilin D [75]. Similarly, miR-579 possesses a significant impact over the damage of host macrophages. The elevated levels of this microRNA can lead to the suppression of Sirtuin 1–Phosphoinositide dependent protein kinase 1 (SIRT1-PDK1) expression and thereby promote the activity of *Mtb* [88]. While the TLR2/MyD88/NF-κB signaling pathway is necessary for the activation of miR-27b, its overexpression can lead to the inhibition of NF-κB expression, thereby affecting the synthesis of pro-inflammatory cytokines. However, via the p53-Reactive Oxygen Species pathway, miR-27b aids in the clearance of *Mtb*. Studies have shown that miR-27b targets Bag2 (Bcl2 associated athanogene 2). This plays a dual role in cellular signaling functioning as both a positive modulator of NF-κB and a negative modulator of p53 signaling [89]. Another feature is the effect of miR-223, where it has been reported that it downregulates the forkhead box O3 (FOXO3). The rate of apoptosis was observed to be reduced where the levels of miR-223 were elevated. Studies have also proven that upregulating FOXO3 can oppose the activity of miR-223 on apoptotic suppression [90]. In addition, Zhu et al. reported that the reduction in miR-18b levels during tuberculosis promoted the expression of its target, HIF-1α, which in correspondence upregulated p38-MAPK and NFκB p65 pathways. The latter raised the synthesis of pro-inflammatory factors, thereby challenging the bacterial survival [91]. This suggests that epigenetic mechanisms play a significant role over apoptosis during tuberculosis. The modulation of host cells via the various conditions discussed above highlights the potential of this area for further research analysis.

## 4. Harnessing Epigenetic Modifications: A Pathway to Host Directed Therapies

*Mycobacterium tuberculosis* employs several mechanisms to combat host strategies, among which epigenetic modifications play the most prominent role. Gaining a comprehensive awareness about these strategies is crucial for the early detection, treatment, and prevention of tuberculosis. It will also provide new insights into the pathophysiology of tuberculosis and contribute to developing more potent vaccines and treatment approaches [46]. Provided the uncontrolled rise in the prevalence of antibiotic-resistant problems, including the paucity of novel antibiotics, it is imperative to discover new strategies to combat the existing issues. According to reports, the encounter between host and pathogen results in chronic immunological changes that would confer survival advantage to the pathogen [92]. Through epigenetic intrinsic heterogeneity, the pathogen can alter the phenotypes of antimicrobial resistance without any mutation of genes [93]. Various mechanisms have been reported, among which phase variation is a significant one. Individual gene expression can be arbitrarily switched to develop a population with a variety of phenotypes that can adapt to varying conditions [94]. Methylation of particular rRNA sites may culminate in antibiotic resistance by preventing antibiotics from attaching to the desired targets [93]. Non-coding RNAs have influence over antimicrobial resistance via plasmids with resistance genes. It is critical to comprehend the rate at which horizontally transferred genes provide advantages or detrimental effects to thoroughly understand the dissemination of such transgenes to the natural microbial community as well as the influence of horizontal gene transfer over their evolution [95].

Host-directed therapy is one such promising strategy to mitigate these issues [19]. HDAC inhibitors are extensively explored in connection to this concept. The use of resveratrol or SRT1720 for the activation of SIRT was observed to alter the inflammatory reactions [96]. HDAC inhibition can lead to the suppression of cytokine release caused by the *Mtb* infection, suggesting the impact of the incorporation of such inhibitors for the therapy [10]. However, further investigations on this study are required to achieve better insights into cytokine synthesis. It is important to have proper information on the cytokines and chemokines responsible for both mycobacterial suppression as well as enhancement [97]. The FDA-approved HDAC inhibitor, suberoylanilide hydroxamic acid (SAHA), has been observed to have an impact over the epigenetic reprogramming within the host macrophages, thereby enhancing the release of IL-1β, which is a pro-inflammatory cytokine leading to the clearance of *Mtb*. It also suppresses the synthesis of IL-10, which is an anti-inflammatory cytokine [59]. Therefore, in the context of HDT, SAHA is considered an immune-augmenting therapy [54]. NFκB signaling is also suggested as a potential target for promoting M1 macrophage polarization by in silico studies [98].

A recently established area of study is the use of miRNAs as novel categories of pharmacological targets for the treatment of different diseases. One apparent strategy is to directly use specific miRNAs that can combat the activity of *Mtb* [35]. Direct miRNA administration can impact the process in different ways, resulting in upregulation as well as downregulation. One instance of this is the administration of miR-223 nanoparticles, which resulted in the transition of phenotypic changes [99]. Positive or negative regulation can be exploited to modify miRNA expression for therapeutic objectives. Innovative approaches use miRNA mimics to obtain appropriate expression or anti-miRNA to prevent inappropriately generated miRNAs [100]. Approaches that can encapsulate miRNAs and transfer them to immune response cells, such as macrophages, are particularly intriguing in the context of infectious disorders. This is because these cells naturally hold the capacity to internalize foreign matters [101]. The miR-29a-3p has been observed to distinguish active and latent tuberculosis, showing its potential in diagnosis [102]. Epigenetic concepts have also significance over vaccine development and mechanisms. Kleinnijenhuis et al. have reported that Bacille Calmette-Guèrin (BCG) stimulates innate immune responses via an interaction incorporating a NOD-2-mediated epigenetic alteration at the level of trimethylation of histone H3 at lysine 4 (H3K4me3). They also reported alteration in the methylation profile of the specific cytokine promotors observed following the BCG vaccination over circulating cells, as well as the suppression of in vitro training effects via methyltransferase inhibitors. These findings demonstrate the possibility of epigenetic reprogramming in human innate immune responses [103].

There exist many hurdles before the proper execution of epigenetic targeted therapy. The development and administration of RNAi is hampered by technical issues such as stability, off-target consequences, immune stimulation, and delivery concerns [104]. It is apparent from reports that the expression of miRNA is variable and heterogenous during the course of the disease due to several factors which include age, gender, and even the platform that aids in profiling [40]. In addition, a serious concern is that epigenetic therapies have the ability to result in unexpected results and may lead to accidental and severe consequences, including adverse reactions, developmental anomalies, and also tumor progression. To mitigate potential hazards and to ensure safe and efficient treatment, extensive investigations are required [105]. Since these epigenetic inhibitors possess limited safety tags, determining appropriate dose regimens is a necessary requirement [106]. Application of multitargeting drugs can be considered as a potential approach to minimize the adverse reactions, drug resistance, and to even optimize the treatment regimen [107]. However, researchers have worked on modifying the chemical makeup of such molecules, thereby attempting to enhance bioavailability and safety [104]. A fundamental element of the WHO’s milestone for 2035 as part of the “End TB Strategy” is the advanced detection of TB via structured and standardized screening. In order to distinguish active and latent tuberculosis, specific biomarkers can be designed and employed. Therefore, the application of miRNAs as biomarkers is crucial [108].

## 5. Discussion

The role of epigenetics in various disease conditions, including tuberculosis, is being explored lately. Numerous methods of survival have undoubtedly been produced by the long-term coevolution of *Mycobacterium tuberculosis* with its human hosts, particularly inside macrophages. Deciphering these strategies will facilitate the formulation of better treatment approaches [109]. Tuberculosis is gaining more significance due to the impressive exploration in the area of epigenetics and how such modulations can lead to the severity of the disease. More extensive studies are necessary to back the insights with scientific evidence. The ultimate strategy of the pathogen is, however, modulating the transcriptional machinery of the host, which includes upregulation or downregulation of specific immune genes, thereby affecting the immune response of the host. Constant research investigations are being proceeded for framing strong conclusions in this context, which thereby brings innovative ideas to tackle this dire disease condition. Unraveling the mechanisms involved in the interactions between the host and the pathogen, such as autophagy, apoptosis, and macrophage polarization, can expand the therapeutic potential of the concept of host-directed therapy. Upregulation of the *Mtb* protein, enhanced intracellular survival (Eis), has been reported to confer resistance against kanamycin. An Eis inhibitor, haloperidol, has been observed to partially recover the kanamycin susceptibility, providing a significant outcome. However, corresponding to the neurotoxic effects of the compound, further studies have to be considered [92,110]. In-depth investigations must be conducted on similar compounds or inhibitors that can suppress the bacterial epigenomic mechanisms. Reports suggest that complementary anti-miRNA can be used to suppress the elevated levels of pro-mycobacterial miRNAs. Similarly, synthetic oligos can be employed for elevating the downregulated anti-mycobacterial miRNAs [42]. An anti-miRNA previously reported against hepatitis C infection, miravirsen, was observed to be effective, which shows the significance of this strategy to be implemented against bacterial infections as well [111]. Drugs like DNA methyl transferase and histone deacetylase inhibitors can address abnormal gene expressions relevant to various diseases. Preclinical research has shown promising potential in this context [105]. Therefore, the practical application of epigenetics targeted therapy against normal as well as drug- resistant tuberculosis involves the need of understanding the epigenetic changes, proper targeting of epigenetic regulators, and implementation of combination therapy. Host directed therapy can enhance immune modulation through targeting specific pathways and personalizing therapies based on individual epigenetic profiles to improve the outcome.

Considering the global prevalence, it is essential to implement efforts on identifying novel therapeutic strategies, potent diagnostic methods, as well as preventive strategies. More advances must be implemented to address the existing limitations in the field of host epigenomics in tuberculosis. The prospects for epigenetics and host-directed therapy in addition to the medications currently available appear promising, as many of the complicated interactions between host and bacterial epigenomics are yet unexplored. Together, these approaches can make differences in confronting this alarming disease.

## Figures and Tables

**Table 1:** An overview of epigenetic modifications induced by *Mycobacterium tuberculosis* and its role in cytokine expression.

Epifactor	Modification/Enzyme	Mechanism	Reference
*Mtb*	miR-146a	Targets TRAF6 and IRAK1, leading to depletion in the levels of TNF-α, IL-1β, IL-6, MCP-1.	[33]
*Mtb* H37Rv	Histone Phosphorylation Histone acetylation	CCR5/ERK regulated modifications elevated the levels of IL-10.	[49]
*Mtb*	HDAC6 HDAC11	Interference in the levels of HDAC6 and HDAC11 can impact the levels of IL-10.	[50]
*Mtb*-Lipomannan	miR-125b	Reduced levels of TNF-α.	[52]
*Mtb*	miR-223	Reduction in the levels of CXCL3, CCL3, IL-6.	[55]
*Mtb*	miR-27a	Aims at IRAK-4 and downregulates TNF-α, IL-6, IFN-γ, IL-1β.	[56]

**Table 2:** An overview of epigenetic modifications induced by *Mycobacterium tuberculosis* and its role in autophagy.

Epifactor	Modification/Enzyme	Mechanism	Reference
*Mtb*	Histone deacetylase 1	ZBTB25 gets incorporated with HDAC1 and Sin3a, repressing the IL-12b transcription and thereby preventing the activation of JAK/STAT pathway.	[66]
*Mtb*	miR-23a-5p	Downregulates the expression of TLR2 as well as the activity of TLR2/MyD88/NFκB.	[70]
*Mtb*	miR144	Targets DRAM2 (DNA- damage regulated autophagy modulator 2)	[71]
*Mtb*	miR-889	Downregulates the expression of TWEAK leading to autophagy inhibition.	[73]
*Mtb*	miR-17	Targets the negative modulators of autophagy like Beclin-1, Mcl-1. Elevated levels of this miRNA inhibit the PKCδ phosphorylation.	[74]
*Mtb*	miR-26A	Targets at Mcl-1.	[75]
*Mtb*	miR-426-5p	Inhibits the fusion of autophagosome and lysosome.	[75]
*Mtb* EIS	H3 acetylation	Elevated levels of IL-10 followed by suppression of autophagy and stimulation of Akt/mTOR/p70S6K pathway was observed.	[57]
*Mtb* BRD4	Histone acetylation	Expression through EGFR signaling leads to suppression of lipid-specific autophagy.	[77]

## Data Availability

Not applicable.
